# Knockdown of the *C. elegans* Kinome identifies Kinases required for normal protein Homeostasis, Mitochondrial network structure, and Sarcomere structure in muscle

**DOI:** 10.1186/1478-811X-11-71

**Published:** 2013-09-23

**Authors:** Susann Lehmann, Joseph J Bass, Nathaniel J Szewczyk

**Affiliations:** 1 Medical Research Council/Arthritis Research UK Centre for Musculoskeletal Ageing Research, University of Nottingham, Royal Derby Hospital, Derby DE22 3DT, England

**Keywords:** Autophagy, C. elegans, Kinome, MAPK, Mitochondria, Muscle, Protein degradation, Proteostasis, Sarcomere

## Abstract

**Background:**

Kinases are important signalling molecules for modulating cellular processes and major targets of drug discovery programs. However, functional information for roughly half the human kinome is lacking. We conducted three kinome wide, >90%, RNAi screens and epistasis testing of some identified kinases against known intramuscular signalling systems to increase the functional annotation of the *C. elegans* kinome and expand our understanding of kinome influence upon muscle protein degradation.

**Results:**

96 kinases were identified as required for normal protein homeostasis, 74 for normal mitochondrial networks and 50 for normal sarcomere structure. Knockdown of kinases required only for normal protein homeostasis and/or mitochondrial structure was significantly less likely to produce a developmental or behavioural phenotype than knockdown of kinases required for normal sarcomere structure and/or other sub-cellular processes. Lastly, assessment of kinases for which knockdown produced muscle protein degradation against the known regulatory pathways in *C. elegans* muscle revealed that close to half of kinase knockdowns activated autophagy in a MAPK dependent fashion.

**Conclusions:**

Roughly 40% of kinases studied, 159 of 397, are important in establishing or maintaining muscle cell health, with most required for both. For kinases where decreased expression triggers protein degradation, autophagy is most commonly activated. These results increase the annotation of the *C. elegans* kinome to roughly 75% and enable future kinome research. As 33% of kinases identified have orthologues expressed in human muscle, our results also enable testing of whether identified kinases function similarly in maintaining human muscle homeostasis.

## Background

Kinases are enzymes that alter proteins and lipids by phosphorylation, the addition of a phosphate group. This modification can influence a protein’s steric structure and cause changes in protein-protein binding affinities and enzyme activities [1]. Kinase encoding genes constitute 2% of the human genome making kinases one of the largest protein families, which is termed the kinome. Kinases also appear to play a major role in modulating cellular processes as roughly 30% of intracellular proteins are phosphate bound at any given time [2]. Accordingly, kinase inhibitors account for a large part of drug discovery programs in the pharmaceutical industry. Roughly 150 inhibitors of 42 well validated kinase targets are currently being tested in clinical trials [3]. These 42 targets constitute only about 8% of the kinome. Although further progress is being made in identifying the function of already well-known kinases in the regulation of specific cellular processes, 50% of the kinome remains largely uncharacterized [3]. Thus, we lack an understanding of the complexity of process modulation by a considerable part of the kinome. In this study, we took a multi-level functional genomics approach to identify kinases required for normal function of individual and multiple processes within a single tissue *in vivo*.

To accomplish this work we employed the soil nematode *Caenorhabditis elegans* as it is a convenient multicellular organism for systems biology research [4]. The *C. elegans* kinome contains 438 kinases which have been assigned to 168 subfamilies [5]. Of these subfamilies, 153 are shared with the human kinome [5]. This conservation suggests that 81% of human kinases have homologues in *C. elegans*. Despite knowledge of these kinase encoding genes, several functional genomic screens looking at the developmental and behavioural effects of RNA interference against each gene in the genome [6,7], and the effort to knock out every gene in the genome [4], the *C. elegans* kinome still appears understudied. A search of the *C. elegans* database, http://www.wormbase.org[8], reveals that only roughly 60% of all kinase-encoding genes have been assigned a genetic or RNAi phenotype.

As several past studies failed to detect a developmental or behavioural effect of RNAi against kinase encoding genes, we studied the effect of kinase knockdown by RNAi on subcellular processes within muscle. We chose muscle as it is a highly regulated, adaptable tissue that responds to environmental inputs such as use and nutrition in a balanced fashion in order to maintain whole body homeostasis. Additionally, identification of new therapeutic targets for modulating muscle homeostasis is desirable as inability to maintain muscle can become a major health concern. Severe wasting of muscle is associated with conditions such as disuse, starvation, several diseases, and inevitably occurs in the elderly [9].

To study the kinome requirement for establishing and maintaining cellular homeostasis, we picked two processes that occur in all cell types, protein homeostasis and mitochondrial dynamics, and one process that is specific to muscle, sarcomere assembly and maintenance. We obtained previously utilized RNAi constructs and examined the effect of knockdown of each kinase upon each process in muscle. We established that 159 kinase-encoding genes, 40% of the *C. elegans* kinome screened [5], appear to influence sub-cellular processes within muscle. Of these 159 genes, 64% appear to be required to maintain homeostasis of fully differentiated adult muscle, 32% appear to be required for multiple sub-cellular processes, and 50% have identified human orthologues [5,10] of which 53 are reported to be expressed in human skeletal muscle [11] (Additional file 1). This quantifies the kinome requirement for normal development and maintenance of a single tissue *in vivo* and assigns RNAi phenotypes to 51 kinases for which no phenotype was previously assigned by genetic or RNAi approaches. Similar to a past study of genes known to influence muscle function [12], we found that individual kinases were most frequently required for proper protein homeostasis and least frequently required for proper sarcomere structure. This suggests that past studies aimed at understanding genomic control of sarcomere structure [13,14] have only begun to uncover the complexity of genomic control of muscle.

To better understand the nature of the kinome requirement for maintenance of muscle homeostasis, we performed epistasis tests with kinases whose knockdown triggered muscle protein degradation against known proteolytic signalling mechanisms in *C. elegans* muscle. While knockdown of individual kinases triggered different types of protein degradation, mitogen activated protein kinase MPK-1 dependent autophagy was triggered in close to half of the knockdowns that triggered degradation. Our results not only contribute to the global understanding of the kinome, they may also lead to the discovery of new therapeutic targets for the modulation of muscle homeostasis.

## Results

### Kinases required for normal protein synthesis and degradation in muscle

Modulation of global protein synthesis, degradation, or both, can lead to either cellular hypertrophy or atrophy. In multicellular organisms these processes must be regulated such that adjacent cells can grow or shrink together. For example, adult *C. elegans* muscle and hypodermis undergo coordinated hypertrophy [15]. Similarly, regulation is required so that one tissue does not receive inordinate nutrients. For example, tumours circumvent such regulation. In man, several extramuscular signals are known to affect protein homeostasis in muscle and are thought to do so via a number of mechanisms, rather than via a single mechanism [16]; however in many cases the transducing signals or mechanisms are not known or are inadequately understood.

**Figure 1:**
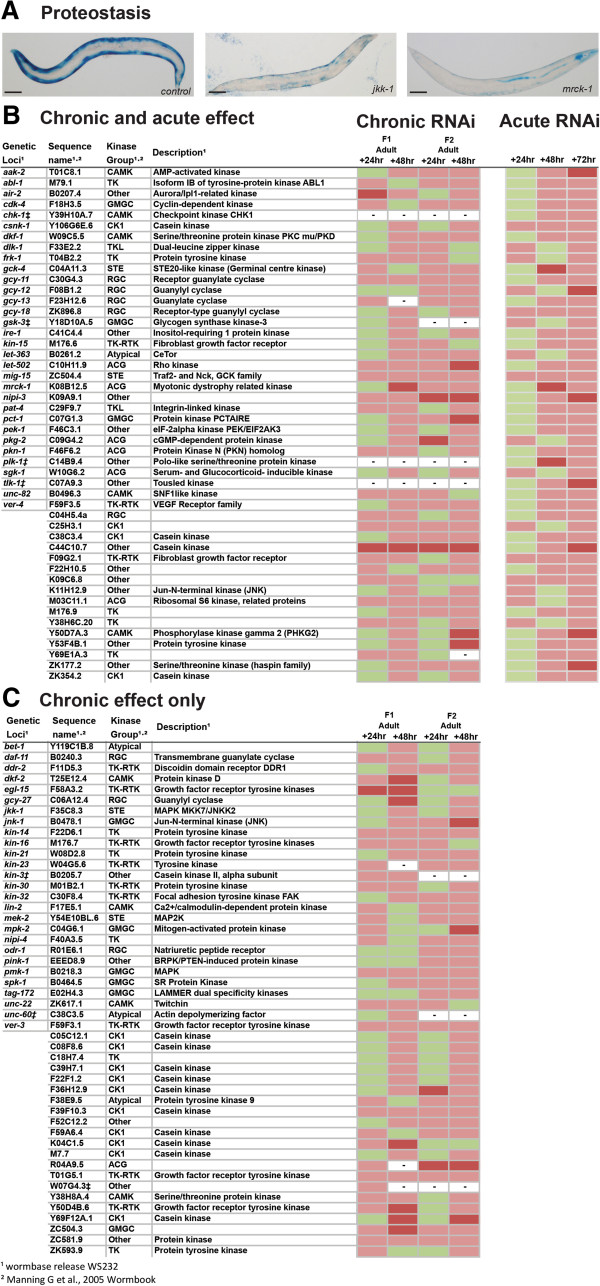
**Kinases that appear to be required for normal muscle protein synthesis and/or degradation. (A)** Sample images of normal β-galactosidase reporter staining (dark blue) in control, non-RNAi treated, PD55 animal (left) and RNAi treated animals showing decreased β-galactosidase reporter staining. RNAi treatments are indicated in the lower right corner. Scale bars represent 100 μm. **(B)** Kinases identified as required for normal lack of protein degradation in muscle. Indicated RNAi treatments were conducted chronically and followed up acutely and scored for decreased β-galactosidase reporter staining, see materials and methods. Displayed are scoring data for each time point (n = 20-30). Green indicates staining not appreciably different from controls. Red indicates that at least 25% of scored animals displayed lighter/diffuse blue or lack of blue staining versus controls. Dark red indicates that at least 50% of scored animals displayed light/diffuse blue or lack of blue staining versus controls. **(C)** Kinases identified as required for normal protein homeostasis in muscle. RNAi treatments were conducted and scored as in **(B)** however the listed treatments produced a defect in the chronic screen but not the acute screen. – Indicates lack of data for a given time point. ‡ Indicates a treatment was classed as producing a lack of progeny in this screen.

To study the effects of altered kinase signalling on cytosolic protein homeostasis, termed proteostasis, we used a well-established *C. elegans* model [17,18]. In this model a myosin heavy chain gene promoter and enhancer drive the expression of a *lacZ* reporter transgene to report on alterations in muscle protein synthesis. As β-galactosidase synthesis stops when animals reach adulthood and the β-galactosidase remains stable for at least 72 hours [17], loss of β-galactosidase in response to acute interventions indicates that degradation, not a reduction in protein synthesis, is occurring in fully differentiated adult muscles. Chronic RNAi knockdown of 96 genes resulted in altered levels of β-galactosidase activity, suggesting that these genes are required for proper regulation of protein synthesis and/or degradation (Figure 1). At least some of these 96 kinases are likely to be required for normal protein synthesis during development, for example EGL-15 which is required for proper muscle development [19], but further experiments are required to confirm to what extent genes identified in the chronic screen may regulate synthesis, degradation, or both. To determine if decreased levels of β-galactosidase activity could be accounted for by increased protein degradation alone, we knocked down the identified genes acutely. We also acutely knocked down genes for which a lack of progeny prohibited full analysis in the chronic screen. Using this approach we identified 48 kinases that appear to be required to prevent abnormal muscle protein degradation (Figure 1). Two of the 48 kinases, *pat-4* and *unc-82*, have recently been shown to be negative regulators of muscle protein degradation in *C. elegans*[15] and two, *gsk-3*[20] and *sgk-1*[21], are known to interact with insulin signalling which is also a known regulator of muscle protein degradation in *C. elegans*[22]. Thus, some of the results from the screen appear to validate our approach. As expected, several kinases without known phenotypes in mutants or in response to RNAi were found to be required for normal protein synthesis and/or degradation or required to prevent abnormal protein degradation (Additional file 1). Lastly, the identification of *ire-1* and *pek-1*, which are well conserved regulators of the endoplasmic reticulum unfolded protein response [23] demonstrates that, as expected, some of the identified kinases act to maintain protein homeostasis not just in muscle, but in most tissues.

### Kinases required for normal mitochondrial network structure

It is now widely accepted that mitochondrial networks are dynamic, undergoing morphological changes to maintain organelle homeostasis or to respond to the metabolic changes within the cell [24]. Advances have been made in determining the mechanisms of mitochondrial organellar quality control; it is however still unclear how mitochondria integrate multiple cellular signals into fission and fusion processes and to what extent mitochondria are self-regulated.

**Figure 2:**
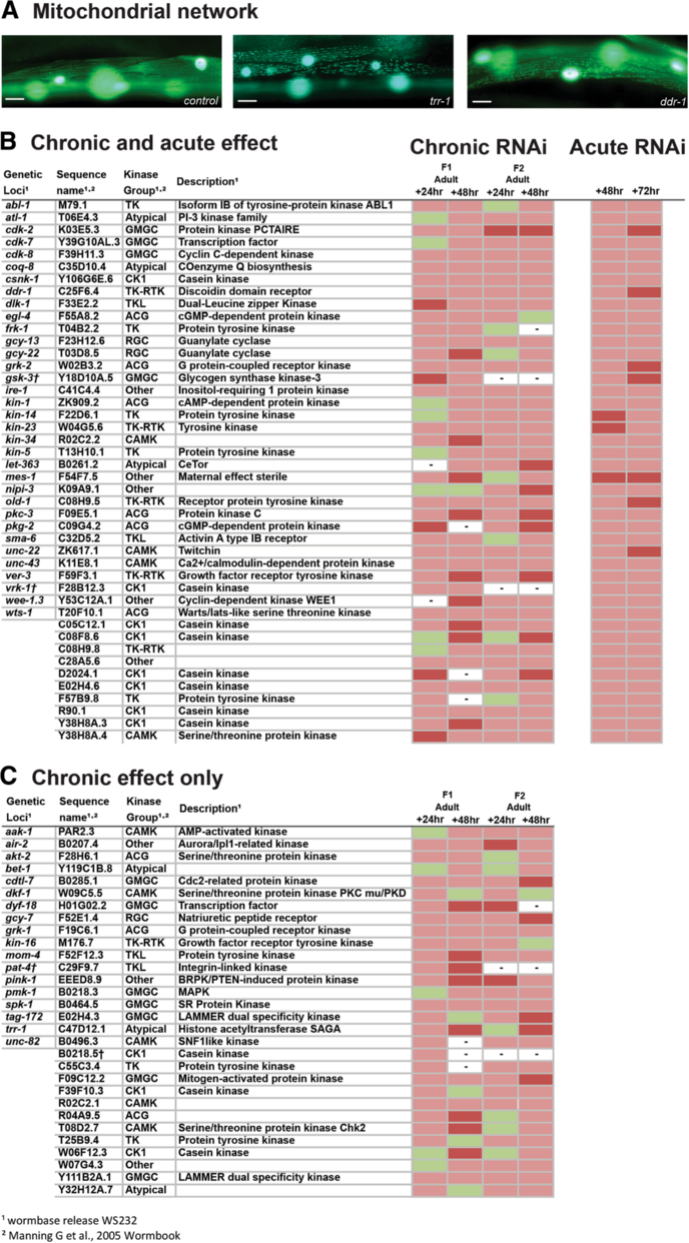
**Kinases that appear to be required for normal mitochondrial network structure. (A)** Sample images of GFP labelled mitochondrial networks, and nuclei (large over exposed circles), showing normal mitochondrial network structure in control, non-RNAi treated, CB5600 animal (left) and RNAi treated animals showing disrupted network structure. RNAi treatments are indicated in the lower right corner. Scale bars represent 20 μm. **(B)** Kinases identified as required for normal maintenance of mitochondrial network structure in adult muscle. Indicated RNAi treatments were conducted chronically and followed up acutely and scored for loss of mitochondrial network organization, see materials and methods. Displayed are scoring data for each time point (n = 20-30). Green indicates networks not appreciably different from controls. Red indicates that at least 25% of scored animals displayed disrupted mitochondrial network structure versus controls. Dark red indicates that at least 50% of scored animals displayed disrupted mitochondrial network structure versus controls. **(C)** Kinases identified as required for normal mitochondrial network structure. RNAi treatments were conducted and scored as in **(B)** however the listed treatments produced a defect in the chronic screen but not the acute screen. – Indicates lack of data for a given time point. † Indicates a treatment was classed as producing a lack of progeny in this screen.

In order to gain insight into the kinome requirement for normal mitochondrial structure in muscle, we examined mitochondrial morphology in *C. elegans* containing mitochondrial localized GFP [25] (Figure 2). Chronic RNAi knockdown of 74 kinase encoding genes induced a fragmented mitochondrial network suggesting that these kinases are required for proper establishment and/or maintenance of the mitochondrial network in muscle. Included within these 74 genes is *pink-1*, which encodes PTEN-induced putative kinase 1, and which when mutated in *Drosophila melanogaster* is known to induce mitochondrial morphology defects in muscle as well as other tissues [26]. Utilizing a *pink-1* knockout allele, see materials and methods, we confirmed that loss of function of *pink-1* results in fragmentation of the mitochondrial network in muscle. To determine if disrupted mitochondrial morphology could be attributed to a kinase requirement for maintenance of the mitochondrial network in muscle we knocked down the identified kinase encoding genes acutely in adults. We also acutely knocked down genes for which a lack of progeny prohibited full analysis in the chronic screen. Using this approach we identified 44 kinases that appear to be required for proper maintenance of the mitochondrial network in muscle (Figure 2). Within this set of 44 genes is *kin-1*, which is already known to be involved in the maintenance of mitochondrial networks [27]. As was the case with the screen for kinases required for protein homeostasis, the identification of known regulators of mitochondrial dynamics appears to support our approach and, as expected, several kinases without known phenotypes in mutants or in response to RNAi were found to be required for normal mitochondrial network structure (Additional file 1).

### Kinases required for normal sarcomere assembly and maintenance

In addition to studying two processes that occur in most tissues, we studied the kinome requirement for a muscle specific process. For this we chose sarcomere assembly and maintenance, as the most recognized function of skeletal muscle is to enable body movement through the contraction and relaxation of many highly organized sarcomeric units. Additionally, as sarcomere structure is conserved from *C. elegans* through higher metazoans, *C. elegans* has become a well-established and validated model for the study of sarcomere structure and assembly [28,29].

**Figure 3:**
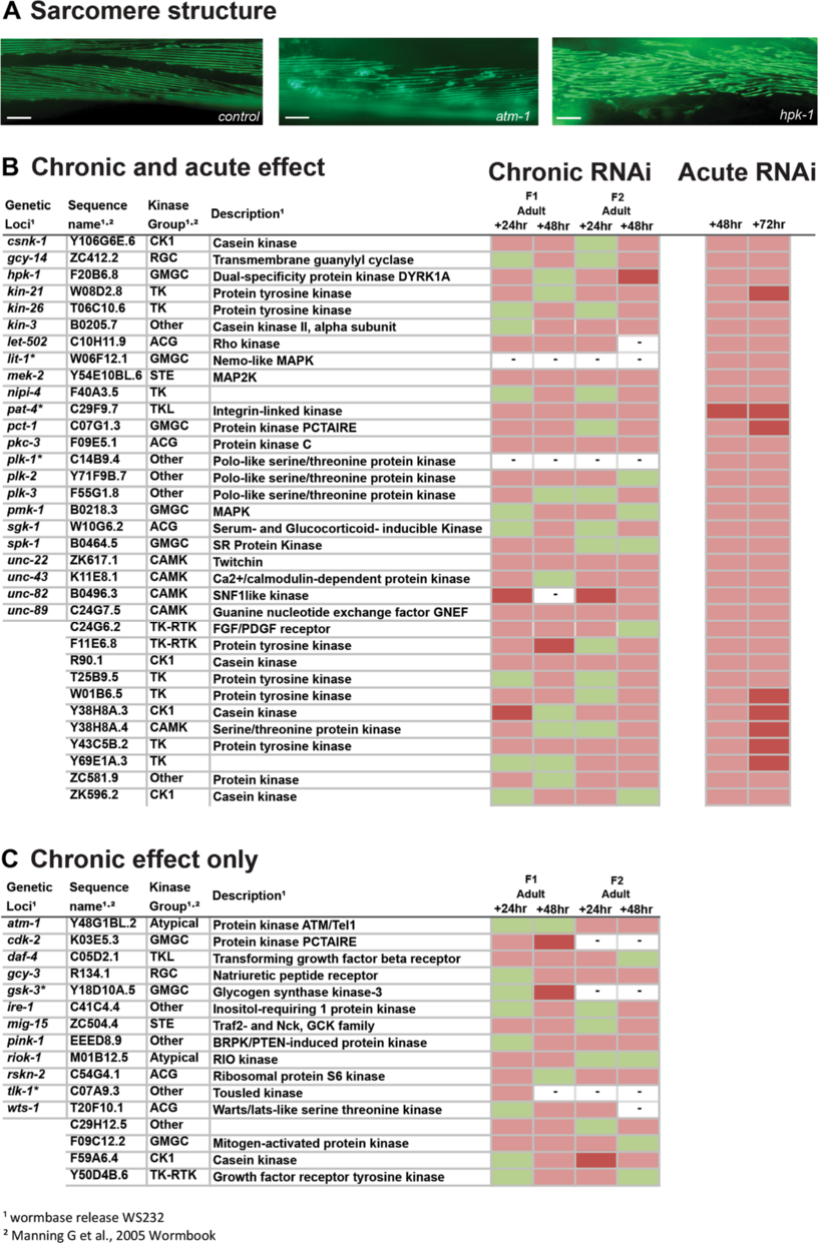
**Kinases that appear to be required for normal sarcomere structure. (A)** Sample images of GFP labelled sarcomeres showing normally aligned sarcomeres in control, non-RNAi treated, PJ727 animal (left) and RNAi treated animals showing sarcomere structure defects. RNAi treatments are indicated in the lower right corner. Scale bars represent 20 μm. **(B)** Kinases identified as required for maintenance of normal sarcomere structure in adult muscle. Indicated RNAi treatments were conducted chronically and followed up acutely and scored for loss of sarcomere organization, see materials and methods. Displayed are scoring data for each time point (n = 20-30). Green indicates sarcomeres not appreciably different from controls. Red indicates that at least 25% of scored animals displayed disrupted sarcomere structure versus controls. Dark red indicates that at least 50% of scored animals displayed disrupted sarcomere structure versus controls. **(C)** Kinases identified as required for normal sarcomere structure. RNAi treatments were conducted and scored as in **(B)** however the listed treatments produced a defect in the chronic screen but not the acute screen. – Indicates lack of data for a given time point. * Indicates a treatment was classed as producing a lack of progeny in this screen.

To examine the effects of kinase knockdown on sarcomere structure, we used animals expressing a myosin heavy chain GFP that localizes to the M-line of sarcomeres [30]. Chronic RNAi knockdown of 50 kinase-encoding genes induced disorganization or tears in the sarcomeric structure (Figure 3) suggesting that these kinases are required for normal sarcomere assembly or maintenance. 12% of these 50 genes are already known to be involved in the regulation of sarcomere structure. For example, *pat-4*, which encodes integrin-linked kinase, and which is part of the muscle attachment complex to the basement membrane [31] and *unc-89 and unc-*22 which encode the structural proteins obscurin [32] and twitchin [33], and which play a major role in the structural integrity of the sarcomeres. To determine if disrupted sarcomere structure could be accounted for by a kinase requirement for normal maintenance of sarcomeres, we knocked down these 50 kinase-encoding genes acutely in adults. We also acutely knocked down genes for which a lack of progeny prohibited full analysis in the chronic screen. Using this approach we identified 34 kinases that appear to be required for proper maintenance of the sarcomeres (Figure 3). As was the case with our other two screens for kinome requirement for sub-cellular processes within muscle, the results of this screen confirm previous observations and identify several genes for which no phenotype was previously assigned (Additional file 1).

### Multiple subcellular defects are more likely to produce a developmental or behavioural phenotype

**Figure 4:**
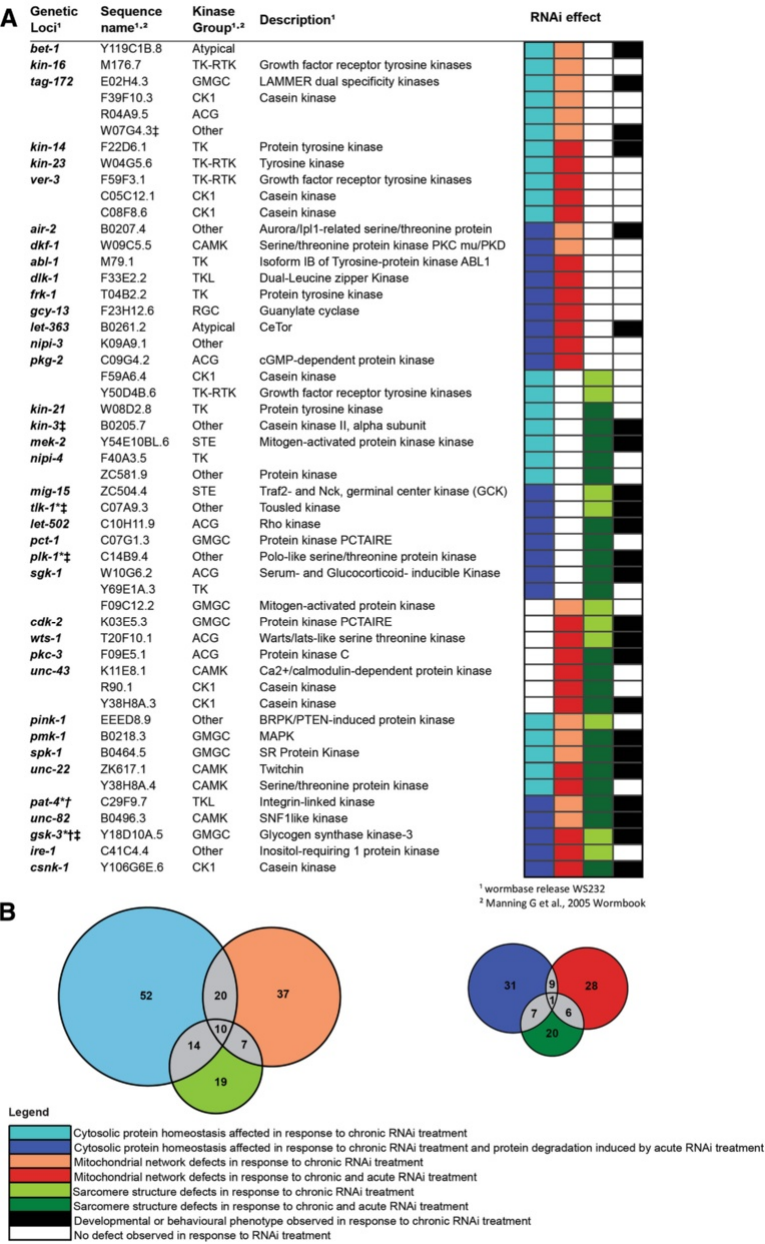
**Kinases that appear to be required for multiple subcellular processes to be normal. (A)** Kinases identified as required for normal muscle protein homeostasis and mitochondrial structure (e.g. kinases identified in both the proteostasis screen and mitochondrial structure screen in Figures 1 and 2), kinases identified as required for normal muscle protein homeostasis and sarcomere structure (e.g. from the proteostasis and sarcomere screens), kinases identified as required for normal mitochondrial and sarcomere structure (e.g. from the mitochondrial and sarcomere screens), and kinases identified as required for normal muscle protein homeostasis and mitochondrial and sarcomere structure (e.g. from all three screens). ‡, †, * Indicates a treatment was classed as producing a lack of progeny in the proteostasis, mitochondria, or sarcomere screen, respectively. Kinases listed on the left relate to colour coded affected subcellular processes and presence of a developmental or behavioural phenotype on the right as described in inset legend. **(B)** Venn diagrams displaying number of kinases which are required for a single process to be normal (data extracted from Figures 1, 2 and 3) and number of kinases required for multiple processes to be normal (grey) during muscle development (left) and/or in adult muscle (right). Colour codes match inset legend. Diameter of circles relate to total number of kinases which are required for this process to be normal.

Comparison of single vs. multiple defects within muscle following RNAi (Figure 4) revealed that kinases appear to be most frequently required to maintain proteostasis and least frequently required for normal sarcomere structure. This result is similar to a past RNAi screen of 159 genes previously known to influence muscle contraction [12]. However, in contrast to this past study, which found an overrepresentation of genes required for normal protein homeostasis, mitochondrial network structure and sarcomere structure, we found that the distribution of kinases required to maintain multiple processes within muscle was not significantly different from a normal distribution (χ^2^, GraphPad Prism). However, there was significant enrichment (p < .05, χ^2^, GraphPad Prism) of developmental or behavioural phenotypes amongst the genes for which RNAi produced defects in sarcomere structure and protein homeostasis and/or mitochondrial network structure (Figure 4, bottom three clusters of genes). Taken together, the results from our study and the past study [12] suggest that past genetic screens aimed at understanding genes regulating muscle function, usually contraction, are more likely to have identified genes that disrupt multiple sub-cellular processes within muscle than genes that disrupt a single process (NB 9 of the 10 kinases we identified in all three screens are well studied). The results also imply that the genomic control of the metabolic functions of muscle, such as protein homeostasis and mitochondrial energy production, are likely underestimated by past studies aimed at understanding the genomic control of sarcomere structure or function [13,14].

### Lack of enrichment of any kinase group amongst kinases identified

We assessed which kinase groups were represented by the genes identified in each of our three screens. A comparison of the number of kinases identified from each group versus the total number of kinases screened in each group revealed no statistically significant (χ^2^, GraphPad Prism) enrichment of any kinase group. This lack of enrichment was observed in each screen and for the total set of kinases identified from all three screens (not shown).

### Epistasis testing of potential degradation-regulating kinases versus known signals

We conducted epistasis tests to gain further functional insight into how some of the identified kinases are acting. Because there are well defined signals regulating protein degradation within *C. elegans* muscle [18] we focused on epistasis testing the genes identified in the protein degradation screen (Figure 1) against the known signalling pathways. In *C. elegans* muscle, presumptive autophagic degradation is controlled by the balance between constitutive, autocrine fibroblast growth factor receptor-Ras-Raf-MAPK signalling [34,35] and insulin growth factor receptor-PI3K-Akt-Raf signalling [22]. As with past studies we used *unc-51*, which encodes Autophagy-related1 (Atg1) [36], mutants to block autophagic protein degradation and we also used mutations in *mpk-1*, which encodes mitogen activated protein kinase (MAPK) [37], and *daf-18*, which encodes phosphatase and tensin homolog kinase [38], to map kinases required to prevent cytosolic protein degradation to activation of the autophagic signalling pathways in *C. elegans*. Proteasomal degradation appears to be controlled by plasma membrane polarization [12] and increased degradation by this system can be observed in response to starvation [17], denervation [39], and neurodegeneration [40]; as with past studies we used the proteasome inhibitor MG132 to block any degradation which required proteasomal activity [39].

**Figure 5:**
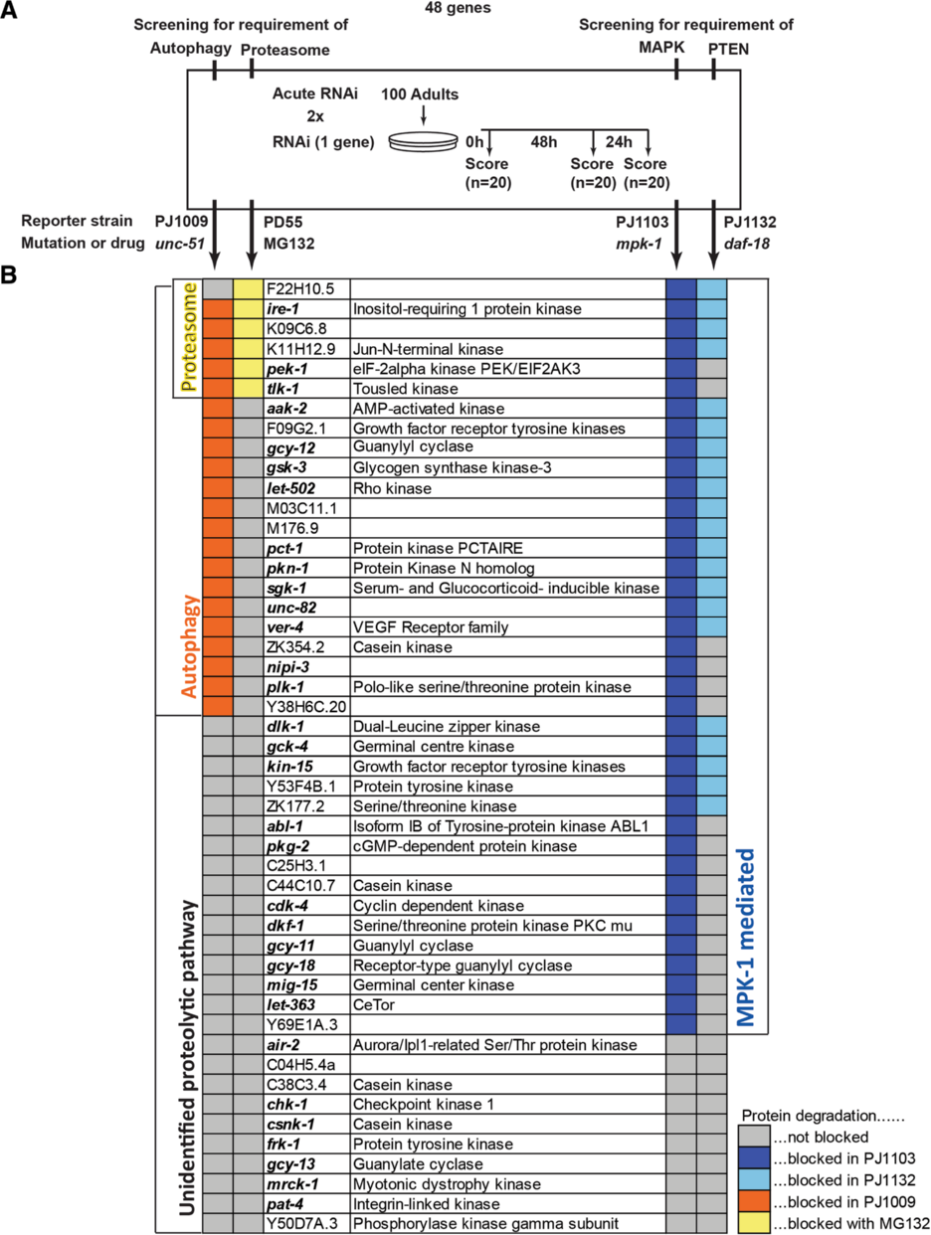
**Epistasis testing of kinases apparently required to prevent protein degradation against known pathways. (A)** The 48 kinases that were identified as required to prevent protein degradation (Figure 1B) were examined for potential interaction with known proteolytic signalling pathways in *C. elegans* muscle [22,34,39]. For these experiments the acute RNAi screen, see materials and methods, was rerun on a control set of PD55 animals (not shown) and on PJ1009 (*unc-51*(*e369*), which has been shown to block autophagic degradation [22]), PD55 treated with MG132 (which has been shown to block proteasomal degradation [39]), PJ1103 (*mpk-1*(*n2521*), which has been shown to block degradation resulting from excessive FGFR or insufficient IGFR signalling [22]), and PJ1132 (*daf-18*(*e1375*), which has been shown to block degradation resulting from excessive FGFR or insufficient IGFR signalling[22]). At least two independent experiments per gene per strain were performed, with a third and/or more run in case of discrepant results. **(B)** Kinase knockdowns identified as requiring autophagy, the proteasome, or another proteolytic system to induce degradation are indicated by the columns on the left. Kinase knockdowns identified as requiring MPK-1 and/or DAF-18 for muscle protein degradation are indicated by the columns on the right. The observation, or lack of observation, of protein degradation caused by knockdown of the indicated kinase (middle columns), in at least 2 experiments, in each of the test conditions is indicated by a colour code for which an inset legend is provided.

**Figure 6:**
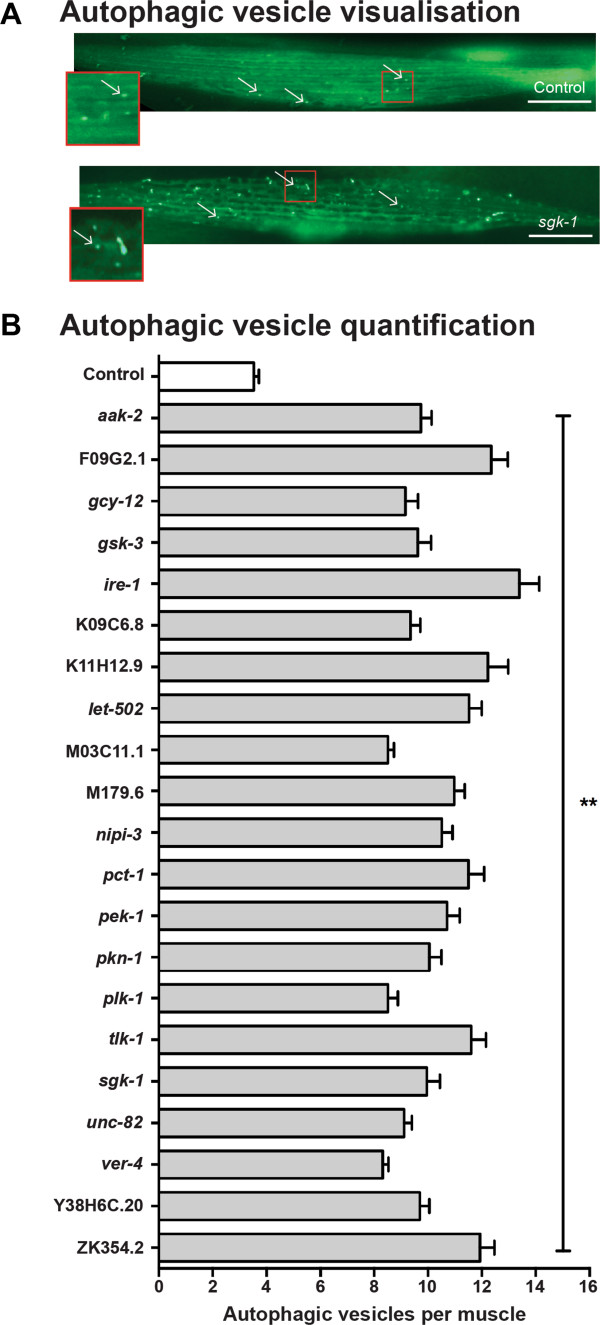
**Increased autophagic vesicles are present in muscles following knockdowns appear to trigger autophagy. (A)** Sample images of GFP labelled autophagic vesicles showing normally low levels in a control RNAi treated KAG146 animal (left) and an experimental RNAi treated animal showing increased vesicles. RNAi treatment is indicated in the lower right corner. Scale bars represent 20 μm. **(B)** Each of the 21 kinase knockdowns that were identified as requiring autophagy to produce increased protein degradation displayed an increase in autophagic vesicles following 24 hours of acute RNAi treatment. Three independent experiments were performed (n = 20 each). Error bars represent standard error of measurement. ** p < 0.0001, one way ANOVA.

For 21 of 48 kinases, β-galactosidase degradation was suppressed in the *unc-51* and *mpk-1* mutant strains suggesting that close to half of the kinases for which knockdown triggered muscle protein degradation are causing increased MPK-1 mediated autophagic protein degradation (Figure 5). To confirm autophagy was triggered by knockdown of each of the 21 kinases, we examined the accumulation of autophagic vesicles in response to knockdown in animals expressing GFP fused to the autophagic vesicle marker LGG-1 [36]. As shown in Figure 6, a significant increase in autophagic vesicles was observed in response to knockdown of each of these 21 genes (p < .0001, one way ANOVA, GraphPad Prism). These results suggest that autophagic protein degradation is the proteolytic mechanism most commonly triggered by decreased expression of an individual kinase. Consistent with this, knockdown of only 6 kinases appeared to trigger proteasome-mediated degradation as evidenced by lack of degradation in the MG132 treatment condition. Two of these kinases, IRE-1 and PEK-1, are well conserved regulators of the endoplasmatic reticulum unfolded protein response which modulates both autophagic and proteasomal degradation. For 21 of the 48 kinases, β-galactosidase degradation was suppressed in *daf-18* animals suggesting that knockdown of these kinases alters signalling in an insulin-mediated pathway. The majority of these kinases also require MPK-1 and UNC-51, which is therefore consistent with past studies of signalling networks controlling protein degradation in *C. elegans* muscle. Included in this group are *sgk-1*[21], *gsk-3*[20] and the gene that encodes AMP-activated protein kinase [41], each of which is already known to be part of insulin-mediated control of protein homeostasis in other species. Additionally, 10 kinases appeared to trigger an as yet unidentified proteolytic mechanism as β-galactosidase degradation upon RNAi knockdown was not suppressed in the mutants or in MG132 treated animals. One of these genes is *pat-4* for which calpains have recently been demonstrated to be the regulated protease [15]. While future study may uncover the details of the other potentially novel mechanisms, it is interesting that autophagy was triggered in response to roughly half of the kinase knockdowns in *C. elegans*.

## Discussion

We have used RNAi to knock down the vast majority of the *C. elegans* kinome to examine the kinome requirement for establishing and maintaining sub-cellular processes within muscle. The use of *C. elegans* and RNAi has allowed us to define a preliminary *in vivo* functional kinome requirement for muscle which currently remains technically and economically challenging to establish in rodents and infeasible in human subjects. The evolutionary distance between the *C. elegans* and the human kinome suggests that not all results obtained in this study will be relevant to man and that there are some important kinases that we have not studied as they do not exist in *C. elegans*. However, our identification of kinases as required for normal subcellular processes in *C. elegans* muscle that are already known to regulate the same subcellular process(es) in mammals, suggests that some, if not many, of our results will be relevant to higher metazoans. Similarly, while our use of RNAi by feeding has allowed us to define a preliminary *in vivo* functional kinome requirement for muscle, our use of this single method will almost certainly have caused us to overlook certain kinases. For example, RNAi by feeding does not produce a reproducible quantitative knockdown from animal to animal [42,43]. Thus, negative results and quantitative differences in defects in response to RNAi against different genes are not interpretable, as it is experimentally challenging to demonstrate quantitative knockdown in the same worm that is scored for subcellular defects and economically not feasible on the scale of the work reported here. Similarly, as the knockdowns occur in the whole animal it is not possible to definitively conclude if it is the knockdown of the targeted gene or another gene that is producing the observed defect nor is it possible to conclude if it is the knockdown in muscle, another tissue, or both that is producing the observed defect. Clearly future studies in kinase gene mutants are required, as are demonstrations of the tissue(s) in which each kinase is required for establishment and/or maintenance of muscle homeostasis. The recent availability of knockouts for most of the *C. elegans* kinome [44] should facilitate such future studies.

Our approach of observing several sub-cellular processes allowed us to analyse differences in the kinome requirement for multiple subcellular processes. For each of the processes studied there appear to be many more specific kinases required than kinases required for multiple processes to be normal within muscle. Thus, it does not appear to be the case that for the majority of the kinome disruption of any one individual kinase results in complete loss of cellular homeostasis. Our data also show that the two general cellular processes, cytosolic protein homeostasis and mitochondrial dynamics, appear to be more frequently affected by kinase RNAi knockdown than the muscle specific process of assembly and maintenance of sarcomeres. This raises the question whether there has been more frequent evolutionary selection for kinases required for general, metabolic processes over those required for other, more specialized cellular processes. Similarly, our observations raise the question whether processes more heavily impacted by kinome knockdown, such as protein homeostasis, are more tolerant of dysregulation without catastrophic failure. If so, this might explain why only 31% of defects in cytosolic protein homeostasis resulted in overall defects in behaviour or development in comparison to 48% of defects in sarcomeres. Together these observations suggest that further analysis of sub-cellular phenotypes or conditional phenotypes should allow assignment of putative function to the entire kinome of *C. elegans*.

Our use of chronic and acute RNAi screens revealed that the majority of kinases appeared to be required for the same subcellular processes during development as in terminally differentiated cells. This observation suggests that a large number of kinases that influence development continue to have an important function in the biology of fully differentiated cells; it may be that cellular regulatory networks are established during development. While it remains to be seen if the kinases regulate development and physiology and do so via identical mechanisms, the observation of conserved effects of knockdown during development and in fully differentiated muscle suggests that candidate drug targets could be selected based upon known roles of genes in the development of tissues of interest.

It remains to be seen if our findings are general features of the kinome in tissues outside of muscle but our identification of kinases that were already known to ubiquitously control protein homeostasis and mitochondrial dynamics suggests that at least some of the kinases identified are likely to affect these processes in other tissues. Additionally, as insulin/insulin growth factor receptor is a general controller of cell size, presumably as the result of MAPK dependent control of the overall rate of translation [45], our observation that functional MAPK is required to produce protein the degradation observed in response to knockdown of most kinases suggests that our observations may be not specific to muscle.

## Conclusions

We identified 159 kinases for which RNAi knockdown induced defects in single or multiple sub-cellular processes in muscle. Some of the identified genes were already known to regulate the distinct sub-cellular processes that we also identified and therefore support the validity of this study. Most of the genes identified are new requirements for subcellular processes examined. For 51 of the identified genes no behavioural or developmental phenotype had previously been reported in functional genomics screens. Thus, our results provide measurable phenotypes to enable more detailed analyses of specific kinases that previously could not be studied *in vivo* and bring the portion of the *C. elegans* kinome that has a known function close to 75%. Lastly, our results may have application to clinical conditions associated with loss of muscle homeostasis. For example, 33% of kinases identified as required for *C. elegans* muscle homeostasis have human orthologues expressed in muscle (Additional file 1). Thus, some of these kinases may serve as the intramuscular transducers of known extramuscular factors that control muscle homeostasis. In this context, it is interesting to note that MAPK appears to be a central regulator of muscle protein degradation in *C. elegans* and the classical MAPK, extracellular-signal regulated kinase (ERK) is known to be functional in adult human muscle [46].

## Materials and methods

### Nematode handling and strains utilized

Nematode strains were maintained and grown at 20°C using *Escherichia coli* strain OP50 as a food source. Strains used were CB5600 (*ccIs4251* I*; him*-*8(e1489)* IV), CC25 (*pink-1* (*tm1779*) II), CC46 (*ccIs4251* I*; pink-1*(*tm1779*) II*; him*-*8*(*e1489*) IV), PD55 (*ccIs55* V), PJ727 (*jIs01; ccIs55* V), PJ1009 (*unc-51*(*e369*), *ccIs55* V), PJ1103 (*mpk-1* (*n2521*) III; *him-8* (*e1489*) *cha-1*(*1182*ts) IV; *ccIs55* V), PJ1132 (*daf-18*(*e1375*) IV; *ccIs55* V), and KAG146 (*kagEx12* (pKG169(*pdyc-1S::gfp::lgg-1*) + pCFJ190(*pmyo-2::mcherry*) + pBSC)). *pink-1* mutant strains were constructed using standard techniques [47], with the presence of *pink-1* homozygotes confirmed by PCR (primers forward 5′ tcattaggatctcgcttgag; reverse 5' agcctcgggcttattaagga).

### Identification and source of RNAi clones utilized

The global list of *C. elegans* kinases [5] was used to search for RNAi bacterial feeding clones previously utilized to determine the effect of knockdown of roughly each gene in the genome upon development and behaviour [6,7]. These identified clones were obtained from Source BioScience (Nottingham, UK). Additionally, a clone against *let-363* was obtained from the University of Colorado [48]. After sequence verifying all positive results from our screen, we identified that previously utilized RNAi constructs were available for 397 kinase-encoding genes, which comprised 91% of the *C. elegans* kinome; clone names beginning with a roman numeral arise from the Ahringer *C. elegans* RNAi library [6] while clones names beginning with an Arabic number arise from the Vidal ORF RNAi library [7] (Additional file 1).

### Quality control of our RNAi screens

**Figure 7:**
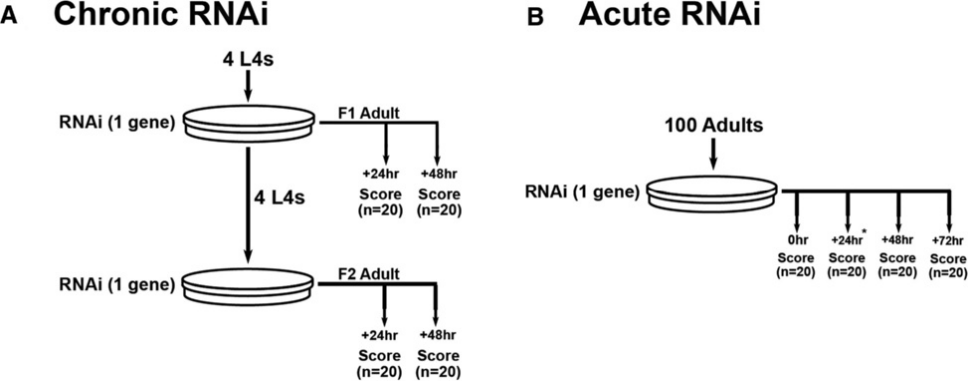
**Systematic identification of kinases required for normal sub-cellular processes in muscle.** Three parallel screens using RNAi feeding clones against 397 kinase-encoding genes were performed. Screens were run in parallel on the same genes, typically 20 per week. In each screen, all genes were assessed for the effect of RNAi upon a single sub-cellular process within muscle (using a different transgenic reporter in each screen). **(A)** All genes were assessed for the effect of chronic, multi-generational RNAi knockdown. PD55 was used for the proteostasis screen, CB5600 for the mitochondrial network structure screen, and PJ727 for the sarcomere structure screen. Upon reaching adulthood, F1 or F2 progeny were observed on two consecutive days using light microscopy after staining for β-galactosidase in the proteostasis screen or epifluorescence microscopy for the mitochondrial or sarcomere screens. **(B)** In each screen, identified genes and treatments that resulted in a lack of progeny were assessed for the effect of acute, single generation RNAi knockdown in age synchronized adults. Adult worms were observed using light microscopy after staining for β-galactosidase in the protein degradation screen or epifluorescence microscopy for the mitochondrial or sarcomere screen (to confirm normal baselines) and then allowed to grow on RNAi plates for an additional 72 hours. Additional measurements were taken 24^*^, 48, and 72 hours after being placed on RNAi plates. ^*^24 hour measurements were only taken in the protein degradation screen.

A schematic of how the three screens were performed is provided (Figure 7). RNAi using bacterial clones grown as described [6] was performed with both chronic and acute RNAi experiments as described previously [12]. A detailed technical description of the strengths, limitations, and caveats to interpretation of results from this screening methodology is available elsewhere [43].

We used RNAi against *unc-112* as a positive control as RNAi *unc-112* has been shown to produce a developmental, behavioural, muscle protein degradation, mitochondrial, and sarcomere defect [15]. Each of the three screens was run in parallel such that the same genes were assessed in the same week, typically 20 genes/week. In cases where the positive control did not provide an abnormal phenotype in each of the screens the entire batch of genes was rerun with an *unc-112* positive control.

We used previously utilized RNAi constructs so that we could directly compare our results for developmental phenotypes with those of others who previously utilized the same RNAi construct as a quality control measure. The false positive rate for developmental and behavioural phenotypes observed in response to RNAi by feeding in *C. elegans* is <1%, thus thresholds were set for ease of scoring. Developmental phenotypes were recorded if at least 20% of worms on the RNAi seeded plate showed a phenotype and also if the same phenotype was observed in both generations, even in fewer than 10% of total worms (NB there was no similar change in threshold in the sub-cellular phenotype analyses). Developmental/behavioural phenotypes scored were: Unc (uncoordinated movement), Rol (rolling movement), Bmd (abnormal body morphology), Dpy (short fat appearance), Pvl (protrusion from the vulva), Rup (rupture from the vulva), Ste (sterile), Egl (egg laying defective), Emb (embryonic lethal) and Gro (long period of development and/or growth arrest). For a complete list of phenotypic results see Additional file 1. Comparison of phenotypes from our study to previously published phenotypes were made using http://www.wormbase.org. Comparison of the developmental and behavioural phenotypes we observed in response to RNAi with past studies that utilized the same RNAi construct revealed a false negative rate of 4% (NB two-thirds of these produced a sub-cellular defect in one or more of our screens), a divergence in phenotype observed of 2%, and a first observation of a phenotype using the RNAi construct for 13% of genes studied. These figures are consistent with another recent study of the efficacy of the method we chose to employ [12] and are an improvement upon the roughly 30% false negative rate typically reported for RNAi by feeding in *C. elegans*. A detailed discussion of why our false negative rate is lower and our first discovery of function rate is higher than past studies as well as other technical details of improving results from RNAi screens can be found elsewhere [43].

All RNAi feeding clones for which a sub-cellular defect in muscle was scored as positive were sequence verified using Source BioScience LifeScience’s Bugs2Bases service or using Source BioSciences’ sequence verified clone service. In order to maximize usable returned sequence data, a combination of three primers were utilised separately for each clone: *C.elegans* RNAi F from the Source BioScience library, forward 5′ ggagaccggcagatctgata, and reverse 5′ ggcctcttcgctattacgc. Sequence data was analysed using 4Peaks (version 1.7.2) software and entered into NCBI *C.elegans* BLAST for confirmation of correct sequence.

### Assessment of proteostasis, proteolysis, mitochondrial network structure, and sarcomere structure via transgenic reporter proteins

Muscle-specific protein homeostasis and protein degradation was assessed using transgene *ccIs55* (*unc-54::lacZ*), with histochemical staining for β-galactosidase activity as described [17]. The protein product of *ccIs55* is continually synthesized throughout development and remains stable (e.g. is neither synthesized nor degraded) in the cytosol for the first 72–96 hours post adulthood in wild-type animals [15,17,22,35,39]. Thus, alterations in β-galactosidase activity observed in response to chronic RNAi indicate alterations in protein synthesis and/or degradation whereas alterations in response to acute RNAi applied to fully developed adults indicate activation of protein degradation alone.

Muscle-specific mitochondria and nuclei were assessed using transgene *ccIs4251*(*Pmyo-3::MitGFP; Pmyo-3::NLS::GFP-lacZ*), with epifluorescence microscopy as described [12]. Note that observations and images were taken of live, non-immobilized animals. This was achieved by capturing images while the animal was still.

Sarcomere structures were assessed using transgene *jIs01*(*myo-3::GFP*) which produces a translational fusion of the full-length MYO-3 (myosin heavy chain A) gene to GFP, with epifluorescence microscopy as described [12]. Note that observations and images were taken of live, non-immobilized animals. This was achieved by capturing images while the animal was still.

### Scoring criteria and procedure for each of the RNAi screens

Sub-cellular phenotypes scored in each of the three screens were: Cytosolic protein content (normal, abnormal), mitochondrial morphology (normal, abnormal), and sarcomere morphology (normal, abnormal); see Figures 1, 2 and 3, respectively, for examples of normal and abnormal sub-cellular phenotypes. In all cases abnormalities deemed minor (e.g. not appreciably different from RNAi control) were scored as normal. Observations were scored as abnormal if defects were observed in at least 25% of worms on the slide (NB this is a 5% increase in threshold from the study [12] upon which this protocol is based). Defects, within an individual worm, were classed using the same thresholds as from the study upon which this protocol is based [12], as follows: i) cytosolic protein content: at least a 30% loss of stain (e.g. intensity viewed as light blue or absent in contrast to dark blue); ii) mitochondrial morphology: loss of at least 30% of the mitochondrial network in at least two muscle cells (e.g. loss of linear networks that was significant enough to be noticeable and different from control animals). iii) sarcomere morphology: at least 2 disorganized or broken arrays of sarcomeres in at least two muscle cells (e.g. loss of linear arrays of sarcomeres that was significant enough to be noticeable and different from control animals). All scoring was done manually using a Nikon H600L microscope.

For chronic RNAi exposures (Figure 7A), a defect had to be observed in any two of the four time points examined to be scored as positive. Genes for which lack of progeny prevented scoring were also scored as giving a defect for the purposes of further examination. Genes that were identified as affecting cytosolic protein content, mitochondrial structure, or sarcomere structure in chronically treated animals were then examined for effects of acute RNAi exposure.

For acute RNAi exposures (Figure 7B), a defect had to be observed at any two time points after introduction to RNAi, with particular attention to progressive loss of cytosolic protein between the 48 and 72 hour time points. The criteria for a positive score at a single time point are identical to those described for the chronic RNAi screens.

For all observations, 4–6 representative images were captured on a Nikon H600L microscope with a Nikon Digital Sight DS-Fi1 digital camera and proprietary software. At the end of all of the screens, these images were used to confirm the results from the screen. Additionally, at the end of all screens all images were reviewed by an independent observer to confirm or correct initial scoring. In cases of a discrepancy between the first and second individual scoring the images the last author manually reviewed the data and the two sets of scoring in order to reach a final scoring.

### Epistasis testing of identified genes against known protein degradation pathways

Mutants and MG132 used for clustering identified genes to known proteolytic pathways/mechanisms were as described [12]. Acute RNAi experiments with these mutants were performed as described [12]. Degradation was scored as in acute RNAi treatments in two independent experiments; in case of discrepancy a third experiment was run.

### Assessment of autophagic vesicles via transgenic reporter protein

Muscle-specific autophagic vesicles were assessed using transgene *kagEx12(pdyc-1S::gfp::lgg-1)*, with epifluorescence. Transgenic animals were synchronised and subject to the acute RNAi protocol with scoring occurring at 24 hours post introduction to RNAi clone or a control clone lacking a targeted sequence. Autophagic vesicles were quantified within the two body wall muscles that visually appeared to contain the greatest number of vesicles. This was repeated in a total of 60 animals per RNAi treatment with 20 animals per experiment and each experiment run three times. Two or three RNAi clones with a separate control clone were analysed at once.

### Statistics

All statistical analysis was undertaken utilizing GraphPad Prism (GraphPad Software, Inc., La Jolla, CA, USA). For assessment of distributions of data being significantly different from a normal distribution, χ^2^ analysis was used. For assessment of tested RNAi clone autophagic vesicle data being significantly different from control RNAi clone, one-way ANOVA with Dunnett’s multiple comparison test was used.

## Abbreviations

C. elegans: *Caenorhabditis elegans*; ERK: Extracellular-signal regulated kinase; GFP: Green fluorescent protien; MAPK: Mitogen activated protein kinase; PI3K: Phosphatidylinositide 3 Kinase; RNAi: RNA interference.

## Competing interests

The authors declare that they have no competing interests.

## Authors’ contributions

SL, JJB and NJS designed the studies and analyzed the data. SL and JJB conducted the RNAi experiments. JJB and NJS conducted the sequence analysis. SL and NJS wrote the paper. All authors read and approved the final manuscript.

## Supplementary Material

Additional file 1 **The following additional data are available with the online version of the paper.** A comprehensive list of genes screened, clones used, and results. Sheet “All genes screened” provides information and results for 397 genes screened including the bacterial clone used in this study, subcellular defects observed as indicated by colour coding (see legend at bottom) and developmental phenotypes observed, known human orthologues along with data on expression in human muscle. Sheet “no clones available” displays all genes for which an existing bacterial clone in the *C. elegans* RNAi library/Ahringer and *C. elegans* ORF-RNAi library/Vidal libraries was not available or for which sequencing suggests it may contain an incorrect target sequence. Click here for file
